# Assembly of multicomponent structures from hundreds of micron-scale building blocks using optical tweezers

**DOI:** 10.1038/s41378-021-00272-z

**Published:** 2021-06-12

**Authors:** Jeffrey E. Melzer, Euan McLeod

**Affiliations:** Wyant College of Optical Sciences, The University of Arizona, Tucson, Arizona 85721 USA

**Keywords:** Optical materials and structures, Micro-optics

## Abstract

The fabrication of three-dimensional (3D) microscale structures is critical for many applications, including strong and lightweight material development, medical device fabrication, microrobotics, and photonic applications. While 3D microfabrication has seen progress over the past decades, complex multicomponent integration with small or hierarchical feature sizes is still a challenge. In this study, an optical positioning and linking (OPAL) platform based on optical tweezers is used to precisely fabricate 3D microstructures from two types of micron-scale building blocks linked by biochemical interactions. A computer-controlled interface with rapid on-the-fly automated recalibration routines maintains accuracy even after placing many building blocks. OPAL achieves a 60-nm positional accuracy by optimizing the molecular functionalization and laser power. A two-component structure consisting of 448 1-µm building blocks is assembled, representing the largest number of building blocks used to date in 3D optical tweezer microassembly. Although optical tweezers have previously been used for microfabrication, those results were generally restricted to single-material structures composed of a relatively small number of larger-sized building blocks, with little discussion of critical process parameters. It is anticipated that OPAL will enable the assembly, augmentation, and repair of microstructures composed of specialty micro/nanomaterial building blocks to be used in new photonic, microfluidic, and biomedical devices.

## Introduction

The miniaturization of existing technology has frequently led to improvements in device performance and efficiency. However, miniaturizing 3D structures with multiple material components and micron-scale or smaller resolution remains a challenge in photonics, electronics, and fluidics. For example, in photonics, designs exist for materials and devices that are difficult to fabricate using existing approaches^1–3^. Working toward addressing these challenges, a variety of techniques have been developed, including grayscale lithography, self-assembly, direct ink writing, and direct laser writing techniques such as two-photon polymerization (TPP)^4–12^. Grayscale lithography can quickly pattern large areas with contoured topography; however, it cannot create complex 3D structures such as overhangs^13,14^. The complexity of structures that can be created by self-assembly has significantly increased over the past few decades through advances in selective functionalization of different regions of particle surfaces with specific chemical linkers such as DNA oligomers^15–19^. Nonetheless, it is challenging to create truly arbitrary (i.e., large aperiodic) 3D geometries entirely using self-assembly. Direct ink writing is typically limited to larger (>10 µm) building block sizes^20,21^. TPP can pattern a photoresist in 3D geometries with a resolution on the order of 100 s of nanometers^22^, but it is not ideal for multimaterial structures. In both TPP and grayscale lithography, multiple development and registration steps and/or special postprocessing techniques are required for multimaterial integration, such as metal plating, atomic layer deposition, or chemical vapor deposition^23–25^. It is also a challenge to use grayscale lithography or TPP to augment or repair existing 3D structures, such as hybrid micro/nanophotonic devices^1,2^. Another common problem in grayscale lithography, direct ink writing, and TPP is shrinkage of the fabricated structure during development when a solvent is removed. While the general structure can shrink by up to 50% in volume, the base is often prevented from shrinking by adhesion with the substrate, leading to warping and deformation of the final structure^26–28^. A further limitation of TPP, grayscale lithography, and direct ink writing is their inability to span a large range of feature sizes; it is advantageous to have small (<100 nm) feature sizes for regions of detail as well as large (>10 µm) feature sizes for rapid filling of volumes. An ideal microfabrication approach would have the ability to pattern in 3D using multiple components in a single platform, enabling the fabrication of structures with spatially varying material properties.

Optical tweezers (OTs), invented by Ashkin and colleagues in 1986^29^, are an alternative manipulation approach for the assembly of micro- and nanoscale structures from building blocks. OTs are noncontact and biocompatible and can manipulate a broad spectrum of particle sizes, morphologies, and materials. In biological applications, they can manipulate living cells and other sensitive materials, such as biochemically functionalized microparticles, without damage^30^. As a result, OTs have found uses in biomechanics^31,32^, tissue engineering^33,34^, and cell sorting^35^. Beyond biological particles, OTs can manipulate metallic nanoparticles as small as 18 nm^36^, dielectric particles on the order of 100 µm using counterpropagating beams^37^, particles of exotic shapes such as cubes, stars, or rings^38^, high aspect ratio nanowires^39^, and composite objects such as core-shell particles^40^. This ability to manipulate preprocessed micro- and nanomaterials across a wide range of sizes is advantageous for fabricating hierarchical 3D structures.

Optical tweezers have received some attention in microassembly studies^41–57^. Small multicomponent structures, typically consisting of two components and fewer than 20 building blocks, have been assembled^43,45,47^. Many studies have also shown the ability to automate the manipulation of large arrays of particles using multiplexed optical trapping in conjunction with image processing and path-planning algorithms^41,51,54^. In addition, relatively large-scale, single-component 3D structures consisting of up to 125 building blocks have been assembled^41,54^. Although there has been great progress in OT-based microassembly over the past two decades, no existing large-scale (several hundred building blocks), multicomponent, 3D structure has been assembled using this approach. In addition, the process efficiency and positional accuracy of OT assembly platforms are not well known. Most OT assembly platforms rely on similar high numerical aperture objectives to tightly focus a laser beam, but the linkage mechanism between building blocks can vary significantly. Different particle linkage approaches include biochemical binding^44–47,58^, photopolymerization^42,52,54^, and engineering of physical interparticle colloidal forces^41,49^.

In this study, we use our optical positioning and linking (OPAL) platform, based on OTs with a biochemical binding mechanism for linking objects, to advance the capabilities of OT microassembly through a detailed analysis of the object placement accuracy, yield, and structure scale. In particular, we investigate the effects of the laser power and biochemical functionalization on the positional accuracy, establishing critical process parameters for optimal performance, and achieve a positional accuracy of ∼60 nm. Using these results, we assemble structures consisting of multiple components with proof-of-concept 3D material variation, culminating in the assembly of the largest 3D, multimaterial microstructure fabricated by any OT-based assembly platform to date, with 448 1-µm building blocks. This structure consists of several hundred 1 µm diameter spheres, which are smaller building blocks than those that have been used in most previous OT microassembly studies. Our approach is robust enough to enable the assembly of such large structures due to the combination of delivery of building blocks using a microfluidic chamber and on-the-fly recalibration to correct for thermal and mechanical drift. Finally, we discuss future approaches that could lead to further improvements.

## Results

**Fig. 1:**
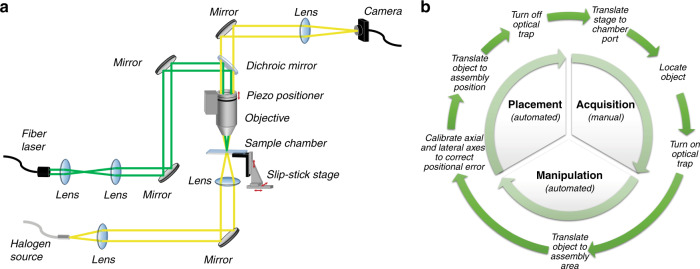
OPAL setup and process. **a** Optical system schematic. Green lines indicate the path of the 1064 nm fiber laser, while yellow lines indicate the path of the halogen lamp white light source. **b** Diagram of the cyclic assembly process. The outer ring illustrates the specific steps within each process stage.

The OPAL platform employed in this study is illustrated in Fig. 1a, with the process flow shown in Fig. 1b. The system components are integrated in a custom LabVIEW interface (Supplemental Figure S1 and Supplemental Video 1, sped up 26×), which provides a semiautomated control platform for the assembly process. The process flow consists of three main steps: object acquisition, manipulation, and placement. Object acquisition is performed manually by the user and entails locating and trapping the desired object. The manipulation and placement steps are fully automated and involve moving the trapped object to the assembly location and placing it at the proper coordinates. During placement, a calibration algorithm updates the assembly coordinate system to correct for any positional errors accumulated during prior movement, ensuring accurate 3D positioning of objects. After placement and trapping laser beam removal, objects remain stationary due to biochemical surface interactions. Neighboring objects have biotin and streptavidin coatings; although this condition on the biological coatings of neighboring objects may appear to limit possible structural geometries, we note that an intelligently chosen order of assembly generally lifts any such restrictions. The order of assembly is typically selected such that sequentially placed objects have complementary functional coatings and are tangentially located while maintaining structural rigidity (see Supplemental Figure S2). Biotin and streptavidin readily form an irreversible and strong noncovalent bond in most environments, generally independent of solvent or pH level. This inability to reconfigure objects after placement necessitates a reliable calibration algorithm such that objects are positioned accurately during the placement step. In the future, a weaker chemical bonding mechanism could be used instead of biotin–avidin to provide the option for annealing structures to improve the regularity; however, this would likely come at the expense of lower bonding strength and structural rigidity. After each successful object placement, the process flow cycles back to the first step (acquisition) to place the subsequent object in the structure.

**Fig. 2:**
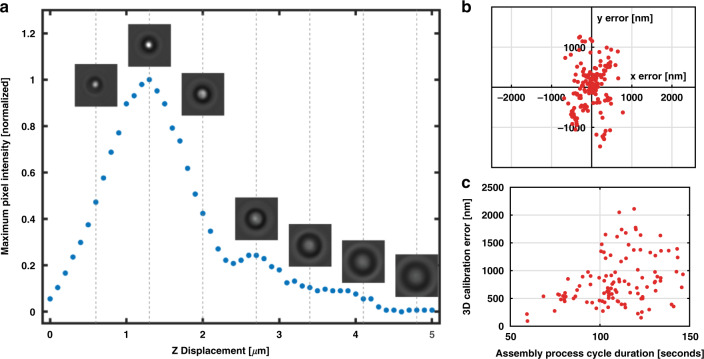
System calibration. **a** Maximum pixel intensity versus stage height during the axial calibration procedure. The inset images show the 1 µm PS calibration sphere at different heights, demonstrating the light-focusing nature of the calibration object. **b** Lateral calibration shift data obtained during an assembly process. **c** For longer fetching and positioning times, the total 3D positional error corrected increases. A longer duration between placements generally indicates that a longer travel distance was required to find a particle, which is positively correlated with the positional error.

The stage calibration procedure that occurs within the placement step recalibrates both the piezo flexure objective nanopositioner and the 3-axis slip-stick stage. Calibration is performed using a 1 µm diameter polystyrene (PS) streptavidin-coated bead that is attached to the biotinylated glass substrate adjacent to the assembly area. Since the sample chamber is illuminated from the bottom, the bead acts as a microlens, and as the elevation of the chamber is varied, there is a position above the sphere where the light comes to a focus (see Fig. 2a). This axial position is the reference for performing the axial calibration. Once the axial position is calibrated, the lateral position is calibrated by cross-correlating the bead with a two-dimensional (2D) Gaussian function to determine the current calibration sphere coordinates in the assembly plane. An initial axial and lateral calibration procedure is performed prior to structure assembly, and the coordinates determined in this process are saved and used as a reference to correct accumulated positional offsets in all subsequent iterations.

Efficiency and accuracy are key process parameters in 3D fabrication. In OT assembly, the efficiency will ultimately be limited by the speed with which objects are translated within the chamber, corresponding to the manipulation step of the assembly process. The translation speed for microscale objects primarily depends on the power of the optical trapping beam, which we previously investigated^59^. Our object translation speeds are restricted to ~200 µm/s due to increasing vibrations associated with the slip-stick stage mechanism above this speed.

**Fig. 3:**
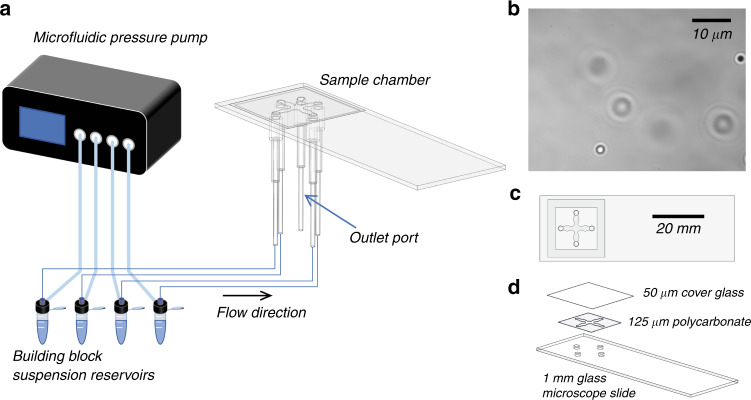
Microfluidic setup and chamber design. **a** Building blocks are loaded into the sample chamber using a microfluidic pressure pump. **b** Typical distribution of building blocks in one of the sample loading channels during assembly. The building blocks shown in this case are 1 µm polystyrene spheres. This distribution corresponds to the initial distribution, as at later times, sedimentation leads to a reduction in the population density. However, in experiments using the microfluidic pressure pump, building blocks can be replenished by flowing additional solution from the building block suspension reservoirs. **c** Top view of one of the sample chambers used in this study. The other type of sample chamber, a 5-port configuration, is illustrated in (**a**). **d** Exploded view of the sample chamber illustrated in (**c**). The chamber consists of three layers that are sealed together using a UV-curing adhesive. The thickness of each layer is labeled in the diagram.

Using our current microfluidic chip, beads are translated ~4000 µm, taking ~20 s per bead. The geometry of the microfluidic chip, illustrated in Fig. 3c, d, is chosen such that building blocks are isolated from the assembly area and each other and thus do not bind spontaneously in solution or in the vicinity of the assembled structure. Particles are held in place for ~2 s to allow for binding, although shorter times may also work. Overall, each full process cycle for the placement of a single object takes ~90 s (~40 building blocks per hour), currently limited by the manual acquisition step, which includes translating the stage and locating a particle. The overall assembly rate is expected to be similar for a wide range of building block sizes. The efficiency is also affected by the placement success rate, or yield. If a biotin–streptavidin bond does not form as expected, then the placement can fail, and another attempt is required.

**Fig. 4:**
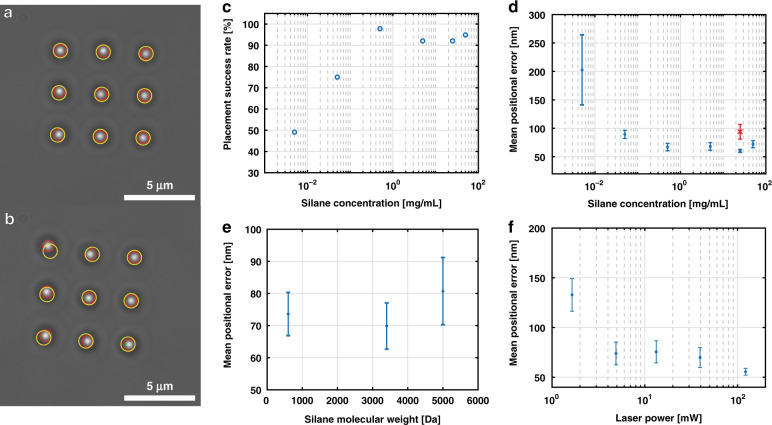
Positional accuracy. **a**, **b** Assembled 3 × 3 grids of 1 µm PS spheres. The red circles coincide with the centroid locations of the observed spheres, while the yellow circles correspond to an idealized grid with uniform spacings. The grid in (**a**) has *σ*_*MAE*_ = 43 nm, while the grid in (**b**) has *σ*_*MAE*_ = 132 nm. The grid in (**a**) was fabricated with application of the calibration procedure, while grid (**b**) was fabricated in the absence of calibration. **c** Placement success rate as a function of silane concentration, using the silane molecular weight (MW) of 3.4 kDa. **d**–**f** Mean absolute positional error as a function of (**d**) silane concentration (MW = 3.4 kDa), (**e**) silane molecular weight (concentration = 25 mg/mL), and (**f**) trapping laser power (MW = 3.4 kDa, concentration = 20 mg/mL). Error bars correspond to the standard error of the mean. **d** Lower silane concentrations lead to increased positional errors, as the reduced surface density of binding groups causes objects to shift upon placement. Data shown in blue are obtained using the calibration process, while the red × data point is obtained without calibration, demonstrating the importance of recalibrating the stage before object placement. **e** Molecular weights between 600 Da and 5000 Da do not show any appreciable effect on positional errors. **f** Reduced laser powers correspond to large positional errors, as the object has a larger mean displacement due to Brownian motion in the weak optical trap.

We quantify the dependence of the placement success rate and positional accuracy on the concentration of biotin-polyethylene glycol (PEG)-silane molecules used to functionalize the substrate. We assemble 3 × 3 grids of streptavidin-coated 1 µm polystyrene spheres on the substrate and compare their experimental positions to an idealized grid with uniform spacing, as shown in Fig. 4a, b. At low biotin coating densities, there may not be a biotin molecule at a particular surface location, leading to reduced placement success rates for functionalization solutions with less than 0.5 mg/mL biotin-PEG-silane molecules, as shown in Fig. 4c. For higher concentrations up to 50 mg/mL, the placement success rate is >90%.

Even if the placement is successful, a low surface coating density can result in positional error (Fig. 4d). This is quantified for each 3 × 3 grid by the mean absolute positional error,

1 $$\begin{array}{*{20}{c}} {\sigma _{MAE} = \frac{1}{N}\mathop {\sum }\limits_i^N \left[ {\sqrt {\left( {x_i^e - x_i^o} \right)^2 + \left( {y_i^e - y_i^o} \right)^2} } \right]} \end{array}$$

where *N* = 9 is the number of objects, $\left( {x_i^e,y_i^e} \right)$ are the experimentally measured coordinates of the *i*^*th*^ sphere, and $\left( {x_i^o,y_i^o} \right)$ are the optimized grid coordinates of the *i*^*th*^ sphere. The experimental coordinates are obtained by capturing an image of the assembled grid and determining the centroid location of each sphere. The ideal, or optimized, grid coordinates are calculated by minimizing the mean absolute positional error using a 5-parameter quasi-Newton optimization algorithm in MATLAB. The fitted parameters include two lateral grid spacings (along *x* and *y*), two coordinates defining the origin of the grid, and the apparent rotation angle of the grid due to a slight rotation of the camera relative to the translation stage axes. The distinction in grid spacings along the orthogonal directions accounts for potential aberrations in the imaging system. As with the placement success rate, biotin-PEG-silane concentrations exceeding 0.5 mg/mL ensure a consistent positional accuracy of 60–70 nm. We also note the importance of the calibration procedure, which ensures accurate positioning of objects throughout the duration of the assembly process. The positional accuracy is measured in the absence of calibration (shown in red in Fig. 4d), which leads to an ~1.5× increase in the mean absolute positional error and an ~5× increase in the standard error of the mean positional error. We expect even further degradation of the uncalibrated positional accuracy when assembling large-scale structures where mechanical and thermal drift become more significant over time, in contrast to the relatively small 9-object grids used for this analysis. This is readily seen in the real-time calibration data shown in Fig. 2b, c, which demonstrate 3D positional errors for a single calibration process of up to ~2 μm.

We also investigate the effect of the molecular weight of the biotin-PEG-silane molecules, which corresponds to the length of the PEG polymer chain. Although we considered that very long chains could lead to positional error and short chains may prevent the biotin molecules from adopting an ideal orientation for binding to streptavidin molecules on the beads, no effect on positional accuracy is found for molecular weights ranging from 0.6–5 kDa, as shown in Fig. 4e. An additional factor affecting the placement accuracy is the laser power, as the trap power correlates with the trap strength. A weaker trap allows for greater object displacements during assembly due to Brownian motion, which can lead to inaccurate positioning. For the 1 µm PS spheres used here, trap powers exceeding 5 mW ensure good positional accuracy, as shown in Fig. 4f. In practice, it is best to use optical powers >50 mW, as this ensures that the trapped sphere can be translated at high speeds without escaping, maximizing the overall efficiency. Although Brownian motion may play a larger role in the assembly of smaller objects, the laser power can typically compensate to ensure that it is not a limiting factor for the placement accuracy. For instance, the trapping stiffness of 100 nm diameter gold spheres was measured to be *κ* ≈ 100 fN/nm/W^36^. This translates to a positional accuracy of $\sqrt {\left( {k_BT} \right)/(\kappa P)} \approx$16 nm for a trapping laser of *P* = 150 mW, where *k*_*B*_ is Boltzmann’s constant and *T* is room temperature, demonstrating that even nanoparticles undergoing Brownian motion could be trapped with precision similar to or better than what we show here.

**Fig. 5:**
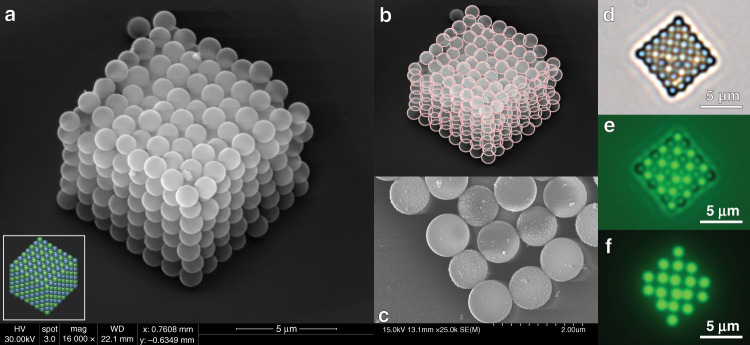
Large-scale microassembly using optical tweezers. **a** Scanning electron microscope (SEM) image of a 3D periodic 8 × 8 × 7 simple cubic lattice consisting of alternating biotin-coated and streptavidin-coated 1 µm polystyrene spheres. Inset: 3D layout of the two components. **b** SEM image from (**a**) with overlaying spheres in red along the front, side, and top faces of the structure, which are used to estimate a mean absolute 3D positional error of 180 nm. **c** High-magnification SEM image of a corner of a 6 × 6 grid of alternating biotin- and streptavidin-coated 1 µm spheres. The streptavidin-coated spheres exhibit a rougher surface. **d**–**f** Optical microscope images of the full structure in (**c**). The biotin-coated spheres are green-fluorescent, while the streptavidin-coated spheres are red-fluorescent. The brightfield image is shown in (**d**), and the fluorescence image obtained using a FITC filter set is shown in (**f**). A mixed modality image (brightfield + fluorescence) is shown in (**e**).

Using the optimized OPAL process parameters, we assemble two-component simple cubic lattices, analogous to the sodium chloride crystal structure. In preparation for SEM imaging, the structures are dried using a liquid carbon dioxide critical point dryer (Tousimis, Autosamdri-815) to prevent damage that can occur during conventional drying. One structure is shown in Fig. 5a, which consists of seven layers of 8 × 8 objects, for a total of 448 objects. This structure takes ~11 h to assemble using our current platform, highlighting the robust nature of our approach, as we place objects accurately throughout long-duration assembly. In Fig. 5b, we show overlaid circles that are used to estimate the positional error. We use a 3D coordinate rotation to estimate the 3D positional accuracy of the lattice from the 2D sphere coordinates in the SEM image (see Supplemental Information), resulting in a 3D positional accuracy of 180 nm. While this is considerably poorer than the 2D positional accuracy achieved while building 3 × 3 grids, we note that the large polydispersity (~10%) of our building blocks greatly influences the 3D positional accuracy when particles are stacked on top of each other. With this in mind, we would expect more uniformly distributed building blocks to achieve better positional accuracy more in line with our 2D results.

Fig. 5c–f show images of a single 6 × 6 layer of spheres, highlighting the differences between the two assembly components. In Fig. 5c, the streptavidin-coated and biotin-coated spheres are easily distinguishable due to the difference in surface roughness. We note that this surface roughness distinction is likely not a result of the larger molecular weight and size of the streptavidin molecules but instead a result of differing microsphere fabrication methods among different commercial vendors. In Fig. 5d–f, optical microscope images show the fluorescence of the biotin-functionalized spheres.

**Fig. 6:**
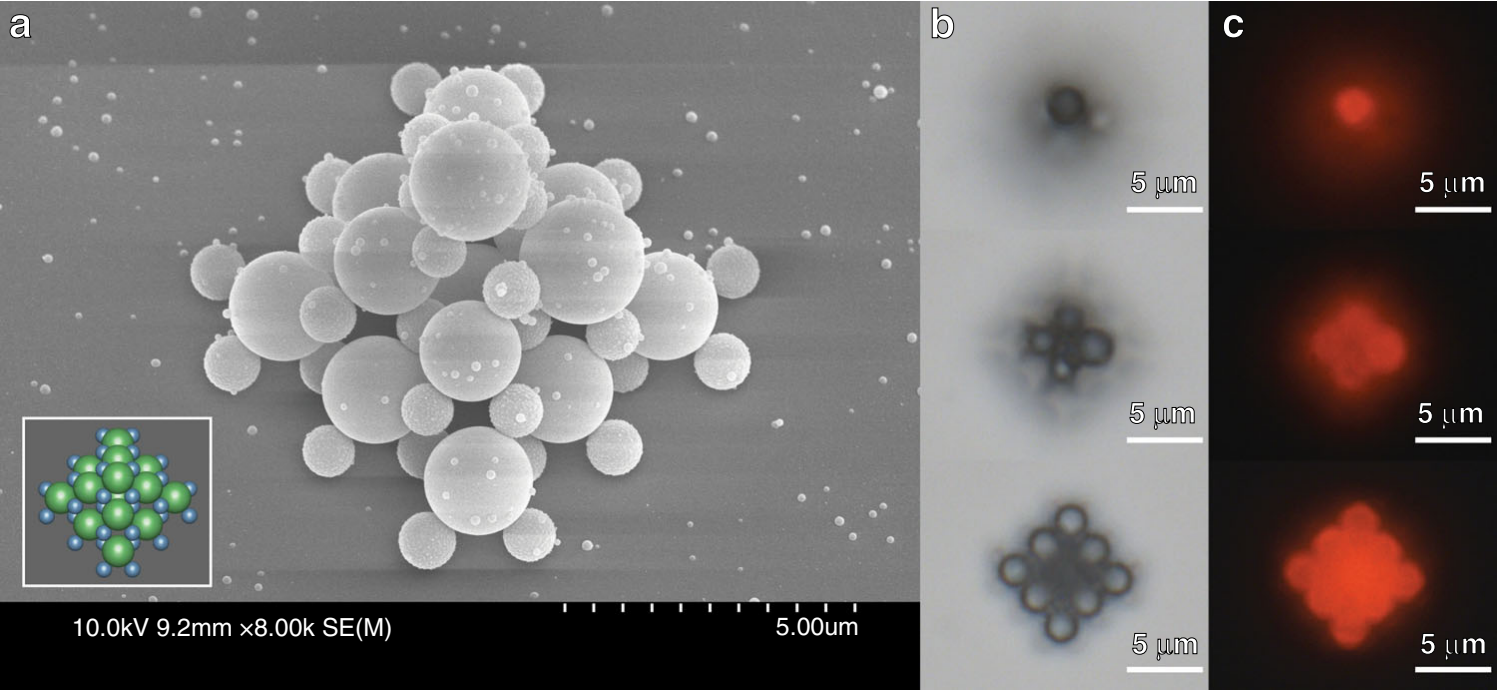
Microassembly of multisized building blocks using optical tweezers. **a** SEM image of a 3D pyramid assembled from streptavidin-coated 1 µm polystyrene spheres and biotin-coated 2 µm polystyrene spheres. The small spots are defects resulting from metal coating for SEM imaging. Inset: 3D layout of the structure, with green spheres corresponding to a biotin-functionalized coating and blue spheres corresponding to a streptavidin-functionalized coating. **b** Series of bright-field optical microscope images at varying elevations to capture the planes that contain the 2 µm PS spheres. **c** Series of epifluorescence microscope images obtained using a Texas Red filter set, captured for the same field of view and elevations as shown in b. These images demonstrate the pink-fluorescent nature of the 2 µm building blocks.

We also assemble a smaller microstructure in the shape of a 3D pyramid with multiple building block sizes (Fig. 6), which demonstrates the flexibility of the platform for the construction of heterogeneous structures. Both building blocks are composed of polystyrene, with the larger (2 µm) spheres being fluorescent with a biotin coating and the smaller (1 µm) spheres having a complementary streptavidin coating to enable object binding. The geometry of this design resembles the cesium chloride crystal structure, except that half of the larger spheres are removed to accommodate the geometric restrictions imposed by the size ratio of the central and corner “atoms.” This yields a coordination number of 8 for the 2 µm spheres and a coordination number of 4 for the 1 µm spheres.

We have shown that an OT assembly platform can efficiently fabricate large 3D, multicomponent structures consisting of several hundred building blocks. We have investigated the effects of the silane concentration and laser trapping power on the assembly positional accuracy, establishing minimum desired values of approximately 1 mg/mL and 5 mW, respectively, for an ~60 nm accuracy. In the future, even larger structures can be built at faster rates with less error by multiplexed optical trapping and full system automation. We note that even though our OPAL approach is currently completely serial, the assembly rate in terms of building blocks per time is the same order of magnitude as that of state-of-the-art multiplexed OT systems^54^. Multiplexing can provide a further efficiency boost and can be achieved by incorporating either a spatial light modulator or a digital micromirror device into the system. Full automation requires automating the building block acquisition step, which could be accomplished using video image processing for microscale particles and/or laser backscatter analysis for nanoscale particles, which may not provide sufficient contrast for brightfield visible image processing. The approach described here only relies on video image processing of a single registration landmark, which can be chosen to be significantly larger than the building blocks used in the structure. We believe that our OPAL platform can overcome critical limitations in current microfabrication technologies, enabling new applications in the fields of photonics and electronics.

## Materials and methods

### Optical setup

The primary components of the optical system include a 1064 nm 30 watt Nd:YAG fiber laser (Spectra-Physics, VGEN-C-30) and a 100×/NA1.1 water-immersion objective (Nikon, MRL07920). The manipulation and positioning of objects is achieved through the combination of a 3-axis slip-stick stage (SmarAct, SLC-1740) and a microscope objective piezo nanopositioner (Piezoconcept, FOC100). Illumination and imaging of the optical trap is performed with a halogen lamp (AmScope, HL250-A) and a CMOS camera (IDS, UI-3480LE-M-G). For experiments requiring laser power measurement (e.g., Fig. 4f), part of the laser power is picked off by a 90 R/10 T beamsplitter in the laser beam path using a photodiode sensor (Thorlabs, S121C) positioned before the objective. By also measuring the power transmitted through the objective in one experiment, the relationship between the picked off power and the laser power after the objective lens is recorded, permitting calculation of the optical trap power at any arbitrary laser power during assembly.

### Sample chamber preparation, building blocks, and microfluidics

The sample chamber used for structure assembly consists of three layers (Fig. 3c, d). The bottom layer, which is also the assembly substrate, is a conventional glass microscope slide with drilled holes that serve as inlet ports for flowing in the multiple assembly objects. The four-port chip shown in Fig. 3c is used for all results, except for the pyramid assembly shown in Fig. 6, for which the five-port chip shown in Fig. 3a is used. The glass substrate layer of the chip is functionalized with a biotin coating *via* silanization with biotin-PEG-silane molecules. The three biotin-PEG-silane molecules used for the experiments in Fig. 4f have molecular weights of 600 Da (Nanocs, PG2-BNSL-600), 3400 Da (Laysan Bio, BIO-SIL-3400), and 5000 Da (Laysan Bio, BIO-SIL-5k). The biotin-PEG-silane molecules are dissolved in dimethyl sulfoxide (DMSO) at a standard concentration of 20 mg/mL unless explicitly varied as described in the main text and Fig. 4 caption. A drop of approximately 5 µL of this solution is deposited on the assembly area for 30 minutes, followed by a rinse with DMSO and deionized water. The second layer of the chamber is a 5 mil (125 µm) polycarbonate spacer layer that defines the walls of the microfluidic chip and is patterned using a CO_2_ laser cutter. The design consists of a central assembly area with multiple channels connecting to the holes drilled in the glass slide. The topmost layer is a No. 000 cover glass (Matsunami). The three layers are sealed together using an ultraviolet-curing optical adhesive (Norland Optical Adhesive, NOA72). Immediately prior to assembly, the chamber is filled with a buffer solution (1X phosphate buffered saline with 0.5% Tween-20 surfactant).

The stock bead suspensions are diluted in buffer solution at a ratio of 1:100. The two beads used in the sodium chloride structure assembly are red-fluorescent streptavidin-coated 1 µm diameter PS spheres (Bangs Laboratories, Inc., CFFR004) and green-fluorescent biotin-coated 1 µm diameter PS spheres (Invitrogen, F8768). The one additional building block used in the pyramid assembly is a pink-fluorescent biotin-coated 2 µm diameter PS sphere (Spherotech, Inc., TFP-2058-8).

Two different microfluidic approaches are used in this study. For the assembly of the large sodium chloride lattice (Fig. 5), ~1 µL of each bead dilution is flowed into its respective inlet port and only a couple millimeters into the chip using a syringe pump. This precise loading procedure ensures that the components do not preemptively mix and bind in the central chamber. One of the holes is left open as an outlet during the inflow process. Finally, the chamber is sealed using a thin layer of polydimethylsiloxane (PDMS). Since this chamber is fully sealed during assembly, no additional building blocks can be introduced. In the assembly of the pyramid (Fig. 6), we do not seal the chamber with PDMS but instead connect microfluidic tubing inlets to the different ports. The building blocks are introduced into the chamber during assembly using a microfluidic pressure pump (Elveflow, OB1 MK3+) while the chamber is in the OPAL setup. By keeping the chip unsealed, a nearly limitless supply of building blocks is available.

## Supplementary information

Supplemental Methods and Figures

Supplemental Video 1
