# Supplementary material for: Assembly of multicomponent structures from hundreds of micron-scale building blocks using optical tweezers
Source: Microsyst Nanoeng. 2021 Jun 12;7:45. doi: 10.1038/s41378-021-00272-z (PMC8433220; doi:10.1038/s41378-021-00272-z)
Supplement: Supplementary file 1 — Supplemental Methods and Figures [file 41378_2021_272_MOESM1_ESM.pdf]

Supplementary Information

Jeffrey E. Melzer[1] and Euan McLeod*[1]

[1]Wyant College of Optical Sciences, The University of Arizona, Tucson, Arizona 85721, USA

*email: euanmc@optics.arizona.edu

Estimating 3D positional error from oblique SEM images

In the coordinate system relative to the substrate (x, y, z basis), we assume the ideal (optimized) particle coordinates are given by:

$$\mathbf{r}_{ijk}^{o} = (x_0 + i\,\Delta x)\,\hat{\mathbf{x}} + (y_0 + j\,\Delta y)\,\hat{\mathbf{y}} + (z_0 + k\,\Delta z)\,\hat{\mathbf{z}} = \begin{pmatrix} x_0 + i\Delta \mathrm{x} \\ y_0 + j\Delta y \\ z_0 + k\Delta z \end{pmatrix}, \tag{1}$$

where i, j, k are used to index the row, column, and layer of a particular particle in the 3D stack, and $x_0, y_0, z_0, \Delta x, \Delta y, \Delta z$ specify the origin of the coordinate system and the particle pitch.

Denote the basis of the SEM image as (u, v, w), corresponding to horizontal, vertical, and depth orthonormal unit vectors. The transformation of coordinates between the bases can be written as,

$$\mathbf{r}_{ijk}^{o'} = A^{-1}\,\mathbf{r}_{ijk}^{o} = \begin{pmatrix} u_{ijk}^{o} \\ v_{ijk}^{o} \\ w_{ijk}^{o} \end{pmatrix}, \tag{2}$$

where the prime (‘) symbol denotes a vector expressed in the u, v, w SEM basis, and the columns of A are the coordinates of the $\hat{\mathbf{u}}$, $\hat{\mathbf{v}}$, and $\hat{\mathbf{w}}$ unit vectors expressed in terms of the $\hat{\mathbf{x}}$, $\hat{\mathbf{y}}$, and $\hat{\mathbf{z}}$ unit vectors. Since the (u, v, w) basis is a 3D rotation of the (x, y, z) basis, it can be expressed in terms of the product of roll, yaw, and pitch rotation matrices for some angles α, β, γ:

$$A^{-1} = \begin{pmatrix} \cos\alpha & -\sin\alpha & 0 \\ \sin\alpha & \cos\alpha & 0 \\ 0 & 0 & 1 \end{pmatrix} \begin{pmatrix} \cos\beta & 0 & \sin\beta \\ 0 & 1 & 0 \\ -\sin\beta & 0 & \cos\beta \end{pmatrix} \begin{pmatrix} 1 & 0 & 0 \\ 0 & \cos\gamma & -\sin\gamma \\ 0 & \sin\gamma & \cos\gamma \end{pmatrix} \tag{3}$$

The experimental coordinates are the optimized coordinates plus some positional error:

$$\mathbf{r}_{ijk}^{e} = \mathbf{r}_{ijk}^{o} + \boldsymbol{\varepsilon}_{ijk} \tag{4}$$

The mean absolute error across all visible particles is:

$$\sigma_{\mathrm{MAE}} = \frac{1}{N_{tot}} \sum_{i} \sum_{j} \sum_{k} \left|\boldsymbol{\varepsilon}_{ijk}\right| \tag{5}$$

Since the (x, y, z) and (u, v, w) bases are both orthonormal, the lengths of any vectors are conserved between the two coordinate systems and $\left|\boldsymbol{\varepsilon}_{ijk}\right| = \left|\boldsymbol{\varepsilon}_{ijk}'\right|$, where $\boldsymbol{\varepsilon}_{ijk}' = A^{-1}\boldsymbol{\varepsilon}_{ijk}$. Because the SEM image is a 2D image and we cannot measure coordinates in the depth (w) direction, the

3D positional error must be estimated based on 2D coordinates alone. If we assume that the unmeasurable error in the w direction is the same as the average of the measurable errors in the u and v directions, then a 2D measurement of positional mean average error should be scaled by $\sqrt{3/2}$ to estimate the full mean average error. So, we can estimate the 3D mean average error as:

$$\tilde{\sigma}_{\text{MAE}} = \sqrt{\frac{3}{2}}\frac{1}{N_{tot}}\sum_{i}\sum_{j}\sum_{k}\left|\boldsymbol{\varepsilon}_{ijk}^{2D'}\right|, \tag{6}$$

where $\boldsymbol{\varepsilon}_{ijk}^{2D'}$ is the positional error vector in the u and v directions:

$$\boldsymbol{\varepsilon}_{ijk}^{2D\prime} = \begin{pmatrix}1 & 0 & 0\\ 0 & 1 & 0\end{pmatrix}\left(\mathbf{r}_{ijk}^{e'} - \mathbf{r}_{ijk}^{o'}\right) = \begin{pmatrix}u_{ijk}^{e}\\ v_{ijk}^{e}\end{pmatrix} - \begin{pmatrix}1 & 0 & 0\\ 0 & 1 & 0\end{pmatrix} A^{-1}\begin{pmatrix}x_0 + i\Delta \mathrm{x}\\ y_0 + j\Delta y\\ z_0 + k\Delta z\end{pmatrix}, \tag{7}$$

where u_{ijk}^{e} and v_{ijk}^{e} are the experimentally-measured positions of the particles in the SEM images, A^{-1} is given by Eq. (3), and $x_0, y_0, z_0, \Delta x, \Delta y, \Delta z, \alpha, \beta, \gamma$ are all fitting parameters whose values are chosen to minimize $\tilde{\sigma}_{\text{MAE}}$ according to Eq. (6). The fitting coefficients we obtained for the SEM image of the sodium chloride lattice (Figures 5a and 5b) are given in the table below.

Parameter	x_0 [μm]	y_0 [μm]	z_0 [μm]	Δx [μm]	Δy [μm]	Δz [μm]	α [°]	β [°]	γ [°]
Value	-0.04	-0.03	-0.04	1.12	1.14	1.03	40.6	29.6	-30.3

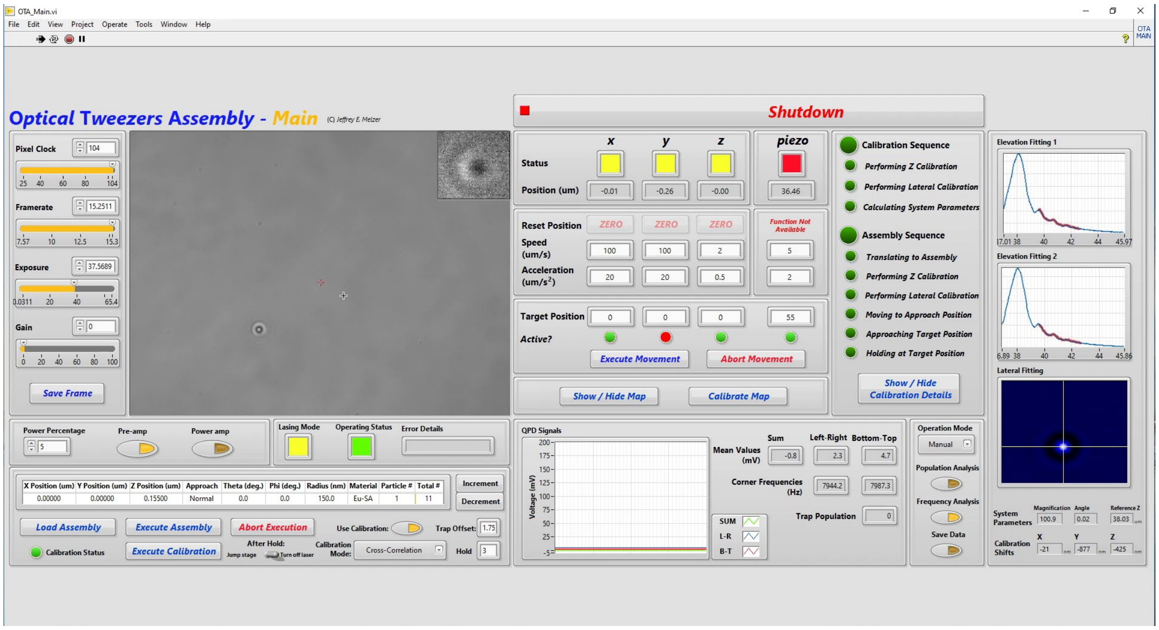

Fig. S1. Front panel of the OPAL LabVIEW interface. The OPAL platform is controlled using a custom LabVIEW code that includes control panels for the microscope camera, optical trapping laser, and nanopositioning stage.

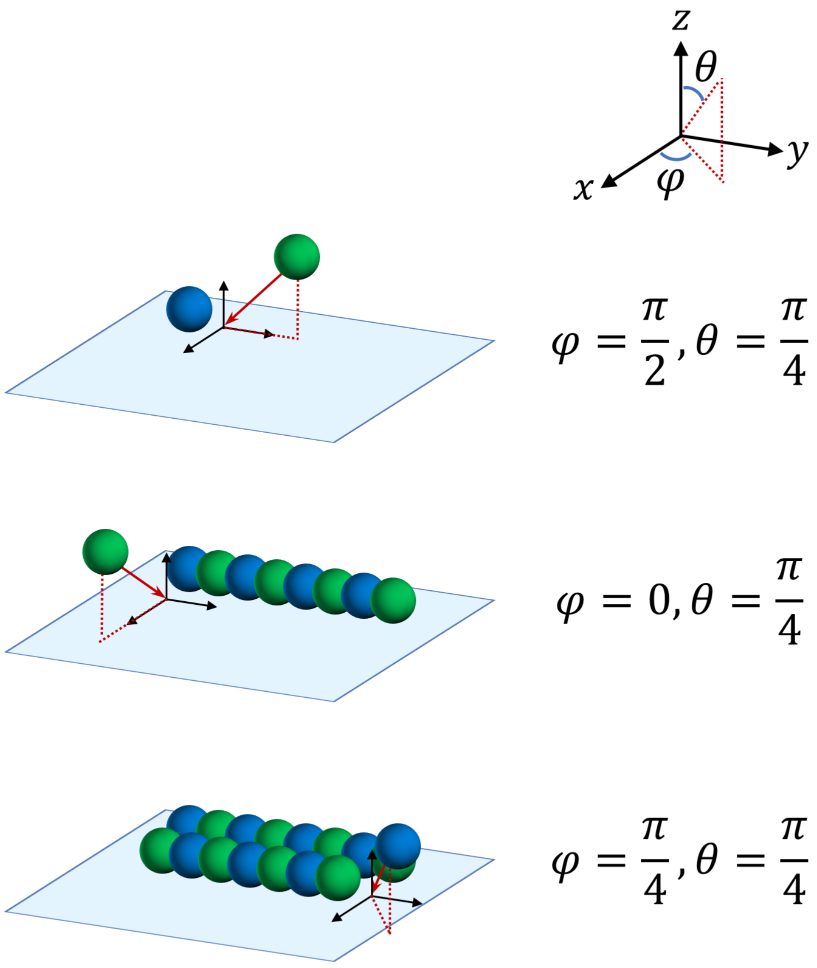

$$\varphi = \frac{\pi}{2}, \theta = \frac{\pi}{4}$$

$$\varphi = 0, \theta = \frac{\pi}{4}$$

$$\varphi = \frac{\pi}{4}, \theta = \frac{\pi}{4}$$

Fig. S2. Assembly order and approach angles. The order in which objects are assembled in chosen using a bottom-up approach that maximizes the number of contact points between neighboring objects, ensuring structural rigidity throughout assembly. The blue and green sphere colors represent complementary surface functionalities, e.g., biotin and avidin.
